# Therapeutic Suggestion

**Published:** 1898-08

**Authors:** W. B. Parks


### THERAPEUTIC SUGGESTION.

BY DR. W. B. PARKS.

Read Before Alumni Association of the Southern Medical College, July 20, 1898.

We have all no doubt read more or less of the history of Mesmer and his mode of inducing sleep as far back as the year 1800. He used the so-called banquet tubs with magnetic ducts filled with soft water and all kinds of ingredients, and armed with iron conductors. At these seances Mesmer appeared in lilac-colored clothes and professed to reinforce the action of the tubs by look and jests, playing upon the harmonica and touching the subject with wand or hand. Mesmer's views still continue in certain circles, though the majority have long since succumbed to the advances of scientific psychology. In this connection it is proper to speak of revived interest in "animal magnetism" due to the researches of Dr. James Braid of Manchester, England. This gentleman in the year 1842 published a work which pretty thoroughly exposed the fallacies of the doctrine of Mesmer, and expounded many of the truths that were entangled therein. He was among the first to employ the term or phrase "animal magnetism," and was the author of the "Hypnotism," though in his day the popular title was "Braidism." Thus we have the different teachings and different theories, and after summing them all up it amounts to about the same in effect. While Mesmer with banquet tubs and all its paraphernalia thought it all was essential to produce the desired effect, Dr. Braid says that placing a bright object in front of the person or subject is all that is necessary to produce the hypnotic condition. The latest and most accepted theory is "suggestive," both normal and abnormal. Dr. Sidis, who is now in the pathological department of the Hospital of New York, has written a very elaborate work on "Suggestive Therapeutics," and from his work I propose to offer as the latest, your subject under consideration. Dr. Sidis says suggestibility is present in what we call the normal state, and in order to know how to reveal it we must know to tap it. The suggestible element is a constituent of our nature; it never leaves us. Before Janet and Binet and many others undertook the study of hysterical subjects, no one suspected the existence of these remarkable phenomena of double consciousness that opened for the new regions in the psycchical life of man. These phenomena were not known, although present all the while; and at times they rose from their obscurity and came to light and obtruded on the attention of the people. They were put down as sorcery, witchcraft, or classed with lying, cheating and deception. The same is true with normal suggestibility. It rarely attracts our attention in trivial things. We do not suspect that the spirit of suggestibility lies hidden even in the best of men.

The present war between the United States of America and the Kingdom of Spain owes its origin to the suggestion of the normal type. Not rationality, but suggestibility, is what characterizes the average specimen of humanity, for man is a suggestible animal. The fact of suggestiblity existing in the normal individual is of the highest importance in the theoretic field of knowledge in psychology, ethics, history, as well as in practical life.

Normal suggestion can be demonstrated in many practical ways, and I have made several experiments similar to those of Dr. Sidis. I select a subject who has not been hypnotized; I ask him to shut his hand tight; then I tell him he can not open it; he tries, with all his might, but can not.

Now, as intimated, we have a duality of the mind; the waking state is denominated as the objective mind. Dr. Sidis claims in the instance of the subject that could not open his hand, that a cleavage or a slight separation took place with the objective mind and that of the subjective mind. This makes hypnotism very simple, for it is only a complete separation of the objective mind from the subjective mind, that produces the phenomena of so-called hypnotism.

As before observed, every individual is susceptible to suggestion at times, and the most susceptible is when suffering from disease, especially of a neurotic type. I would urge upon you, and all other physicians, if you have not, to investigate this great subject of hypnotism and therapeutic suggestion, for suffering humanity calls on us, not only to relieve them of their physical ailments, but of their morbid mental conditions that perchance may be melancholy hysteria. A great therapeutic agent is at our very door; shall we avail ourselves of the great advantages that it holds out to us?

A word about the Southern Medical College. As intimated in the first part of this paper, suggestion not only plays its part in social, political and economical affairs, but silently and surreptitiously, suggestion, not of a normal type, but of a hypnotic form as with the magic wand of Mesmer himself, wielded the potent force and said two colleges are one.

